# Technology-Enhanced Learning in Medical Education Collection: Latest Developments

**DOI:** 10.12688/mep.19856.1

**Published:** 2023-10-20

**Authors:** Derek Choi-Lundberg

**Affiliations:** 1 Tasmanian School of Medicine, University of Tasmania, Hobart, Tasmania, 7000, Australia

**Keywords:** Technology-enhanced learning, web 2.0, e-portfolio, simulation, web conferencing, flipped classroom, interprofessional learning, team-based learning, generative artificial intelligence

Technology-enhanced learning (TEL) refers to learning activities and environments that are potentially improved or enhanced with information and communication technologies ( Shen & Ho, 2020; Wasson & Kirschner, 2020). TEL may be implemented in face-to-face, distance/remote and blended or hybrid modes; in various environments such as online, classrooms, workplaces, communities, and other built and natural environments; include a range of learning designs and pedagogies/andragogies; involve synchronous and asynchronous interactions amongst students, teachers, workplace staff and clients, and/or community members; and delivered with the support of various technologies ( Wasson & Kirschner, 2020). To date, the Technology-Enhanced Learning in Medical Education collection, part of *MedEdPublish*, has received submissions relating to several technologies to support learning, including web conferencing, web 2.0, e-textbooks, e-portfolios, software, generative artificial intelligence, simulation mannequins and wearables for point-of-view video, often in combination. Learning designs included flipped classroom with interactive case discussions ( Imran *et al*., 2022), e-portfolios ( Javed *et al*., 2023), didactic teaching followed by demonstrations of clinical skills on a simulation mannequin ( Zwaiman *et al*., 2023), interdisciplinary case discussions to promote interprofessional learning ( Major *et al*., 2023), patient panels to share narratives and perspectives ( Papanagnou *et al*., 2023), and team-based learning ( Lee & Wong, 2023). In the four papers that included evaluation, participant reaction (feedback on learning activities) and/or learning (self-reported through surveys, with pre- vs post-training comparisons or at different timepoints during learning) were reported, corresponding to levels 1 and 2 of the commonly used outcomes-focused Kirkpatrick model of evaluation ( Allen *et al*., 2022). Two papers focused on the work of health professions educators, including conducting the nominal group technique, a qualitative research method, via web conferencing ( Khurshid *et al*., 2023); and using ChatGPT to assist with various medical education tasks ( Peacock *et al*., 2023).

Imran and colleagues (2022) contributed to the growing body of literature on continuing professional development (CPD) delivered through collaborations amongst institutions across high, middle and low-income countries (HICs and LMICs) ( Hill *et al*., 2021). They used web conferencing for post-graduate training and CPD in child and adolescent psychiatry in Pakistan, with the program collaboratively developed and adapted during delivery by experts from the United Kingdom, Belgium and Pakistan, as part of the World Psychiatric Association Volunteering Program. Thirty participants including postgraduate residents, early career psychiatrists and senior psychiatrists from tertiary care mental health centres across Pakistan attended nine weekly sessions with flipped classroom learning design. Pre-readings were assigned from the World Health Organization’s *Mental Health Gap Intervention Guide* and an international e-textbook. Online web conference sessions were delivered to two groups of 15 participants each and utilised breakout rooms. These synchronous sessions included a summary of the topic presented using PowerPoint slides, question and answer time, and a one-hour interactive case discussion prepared by a trainee and chosen in collaboration with senior trainers. Asynchronous communication amongst participants and local senior trainers was facilitated with web 2.0 (WhatsApp). Feedback from participants was collected after each session, and pre- and post-course evaluations were completed by nearly all participants. Satisfaction was high (4.7 out of 5) and self-reported confidence in knowledge and skills in child and adolescent mental health improved from 2.8 pre-training to 4.0 immediately post-training (average across 10 questions) on a 5-point Likert scale. Participants were satisfied with English as the language of instruction, although the authors note that local language would provide cultural nuance ( Imran *et al*., 2022). The first and second authors of this paper were both from the LMIC, which is rare in LMIC-HIC collaborative CPD publications ( Hill *et al*., 2021). Utilising web conferencing instead of international travel reduces cost and time investments and is more sustainable for the planet and the education collaboration. While challenges in LMIC-HIC collaborative CPD include limited technology and other resources in the LMICs and differences in languages, cultures and healthcare systems, attention to long-term relationship building and local contexts, and genuine collaboration with LMIC team members playing active and leading roles can promote successful outcomes ( Hill *et al*., 2021), as achieved by Imran and colleagues (2022). Factors leading to successful CPD for physician knowledge and performance and ultimately patient outcomes include more interactivity, a variety of pedagogies, longer duration and more exposures, small groups from a single discipline, and a focus on content relevant to the participants ( Cervero & Gaines, 2015), which Imran and colleagues’ study exemplifies.

Interprofessional education (IPE) enables health professions students to learn with, about and from each other to improve patient-centred care and collaboration, ideally across the pre-licensure degree program and with learners at similar stages of development of knowledge, skills and professional identity, with learning activities ranging from exchanges, observations, case-based problem solving, simulations and practice ( Shakhovskoy *et al*., 2022). Major and colleagues (2023) used two-hour web conferences to enable interdisciplinary case discussions amongst pharmacy and medical students during the COVID-19 pandemic, and compared outcomes to eight hours of in-person home visits under supervision of a geriatrics physician or nurse practitioner pre-pandemic, with both formats intended to meet IPE learning outcomes, including interprofessional communication, teamwork, values, roles and responsibilities, through promoting dialogue and reflection. Web conferences using the zoom platform included didactic elements and discussion of three cases, with two to five pharmacy and medical students guided by a preceptor, followed by students working collaboratively on a case, ending with a debrief on their teamwork. Self-assessment of interprofessional knowledge, skills and attitudes were collected pre- and post- learning activities with a modified Interprofessional Collaborative Competency Attainment Survey (ICCAS) with 10 items on a 5-point Likert scale and open response items. Web conference vs in-person formats and pharmacy vs medicine student outcomes were compared, with a total of 459 (84%) responses. Medicine and pharmacy students had increased scores on all ten items after both types of learning activities compared to before, with pharmacy students reporting greater gains from the web conference compared to the in-person format on nine of ten items, whereas medicine students had no significant differences in gains between formats. Thematic analysis of open-response items indicated more comments about interprofessional perspectives but fewer relating to socioeconomic status and the health care system with the web conference compared to the in-person format, likely relating to the intentional construction of cases for the web conferences enabling interprofessional collaboration opportunities, compared to opportunities to observe clients in their homes and hear first-hand their experiences in the in-person format. The design of Major and colleagues’ (2023) web conference IPE aligned with many characteristics identified by Maddock and colleagues (2023) for successful IPE in non-clinical environments, including spending time together in a safe learning environment, clinical cases designed for interdependence so each profession could offer unique knowledge to collaboratively solve the problems presented, and debriefing to consider teamwork and communication.

There is increasing interest in the potential for wearable technology (wearables) in education, including head-mounted virtual or augmented reality headsets or smart glasses, upper limb-mounted devices such as smart watches or gloves, or mobile phones attached to the body ( Almusawi *et al*., 2021). Aligning with this last approach, Zwaiman and colleagues (2023) provide a preliminary evaluation of web conferencing for demonstrating airway management and resuscitation skills on a simulation mannequin using wearable technology (a chest-mounted smartphone) to provide facilitator viewpoint simultaneously with a laptop webcam for mid-distance observer viewpoint to second-year medical students in Canada for distance learning necessitated by the COVID-19 pandemic. The single one-hour web conference was attended by 268 students and run by three facilitators, one with the chest-mounted smartphone demonstrating clinical procedures, one monitoring zoom chat, and one to provide technical and equipment support. Demonstrations were preceded by descriptions of management of trauma patients with fully or partially obstructed airways. Student perceptions were collected via a survey and course evaluation form, although response rates were poor. Five-point Likert scale questions indicated students felt learning objectives were clear (4.5, with 5 = strongly agree) and achieved (4.4), although there was only weak agreement that the online format did not impede learning (3.5). Themes from open-response feedback included appreciation of the dual first-person facilitator and observer viewpoints, some issues with camera instability and positioning (but zoom chat feedback enabled rapid correction of the latter), and requests for greater interactivity and each demonstration to be linked to a clinical scenario. This approach enabled time-efficient demonstration of clinical skills to hundreds of students in an emergency remote teaching circumstance, but students and the authors agreed that this cannot replace in-person skills training, which would normally involve 6–8 students per mannequin practicing skills under supervision of one facilitator in a simulation centre ( Zwaiman *et al*., 2023).

Lee and Wong (2023) compared various learner characteristics and perceptions across team-based learning (TBL) activities delivered online or face-to-face (F2F). TBL is a flipped-classroom approach that includes pre-class learning materials followed by a live class incorporating individual and small group readiness assurance tests (iRAT and gRAT) to promote accountability for engaging with pre-class resources, followed by a clarification phase and discussion facilitated by the teacher requiring application of knowledge, such as to clinical cases. Previous studies have generally found teacher and learner attitudes towards TBL to be positive, with improved learning outcomes compared to didactic approaches ( Reimschisel *et al*., 2017). In Lee and Wong’s study (2023), the main instruction method for the first year preclinical phase of the Duke-NUS graduate medicine course is TBL, which was delivered fully online using web conferencing (zoom) for four months, followed by another four months in hybrid mode with half the cohort attending in person and the other half online via zoom (teams alternated attendance mode weekly) with teachers facilitating online, due to social distancing requirements in response to the COVID-19 pandemic. Breakout rooms in zoom were used for the gRAT phase. Four sets of surveys were completed across eight months, two during the online-only period, and two during the hybrid period, with participants (68% of the cohort) asked to focus on the F2F classes in their survey responses for the hybrid period. Validated instruments were used to measure elements of self-determination theory (SDT: autonomy, competence, relatedness and motivation); various characteristics including curiosity, resilience and growth mindset; meta-cognitive and self-regulated learning strategies; and outcomes including engagement and self-perceptions of learning. Competence, need satisfaction of SDT and perceived learning were higher F2F compared to online, while all other measured variables did not differ. 59% preferred F2F, 12% online, and 29% had no preference for delivery mode of TBL. Qualitative analysis of open-response comments comparing online and F2F modes at the final timepoint suggested small-group interpersonal interactions and discussions were easier in-person with fewer distractions from learning than online.

A Practical Tips article from Javed and colleagues (2023) provide suggestions for implementing electronic portfolios (e-portfolios) in undergraduate dental and medical programs to support programmatic assessment. Programmatic assessment seeks to optimise assessments for learning and high-stakes progression decisions; principles include that each assessment task is designed for learning through provision of meaningful feedback, fall on a continuum of low- to high-stakes, various assessment methods are implemented and aligned with intended learning outcomes to determine their attainment, results and feedback are discussed with the learner at intermediate timepoints prior to high stakes decisions that are made holistically and transparently, and gradually increase learners’ accountability for their learning ( Heeneman *et al*., 2021). E-portfolios provide a technology platform for students, trainees and professionals to collect, organise and select evidence to demonstrate their learning longitudinally. Feedback on e-portfolios from self-reflection, peers and teachers including scaffolding and coaching promotes self-directed learning ( Beckers *et al*., 2016), and e-portfolio platforms enable teachers to assess student learning and evaluate program outcomes. The 12 tips from Javed and colleagues (2023) include conduct a needs assessment prior to implementation; involve all stakeholders in development and evaluation including students, graduates, teachers, administrators, IT staff and future employers; select a suitable e-portfolio platform and integrate with the learning management system; determine formative and summative assessment purposes, processes and timepoints, supported by assessment rubrics and aligned with intended learning outcomes and curriculum; train staff in theory, pedagogy and technology to support e-portfolio use and assessment; start students on their e-portfolio journey in first year with orientation to and training in processes and purposes; provide computer labs and internet access for students to ensure equity of access; provide ongoing training, guidance and support for students and staff to promote effective use of and feedback on portfolios; close the loop on feedback through students reflecting on peer and staff feedback and responding through improving their e-portfolio with subsequent assessment; and follow student progress and celebrate successes. The e-portfolio may support development of skills in reflection, self-directed learning, digital literacies, communication, collaboration, professionalism and transfer of learning ( Javed *et al*., 2023), and may be continued in post-graduate training and during careers for continuing professional development.

A Practical Tips article from Papanagnou and colleagues (2023) provides suggestions for delivering patient panels for pre-clinical medical students via web conferencing, with the COVID-19 pandemic providing the impetus for change from in-person to online delivery. Advantages of web conferencing over in-person include eliminating the need for a large venue and patient transport, supporting patient psychological safety with private chat or SMS communication and the option to turn off video and/or audio to leave the conversation temporarily, and enabling greater student participation through asking questions in chat. Patient panels enable a small number of patients to share their experiences and views on well-being, illness and the health system with a large number of students, who may develop empathy, communication skills and appreciation of patient perspectives. Web conferencing additionally facilitates sharing photos or videos of patients in their environment to enhance student understanding of their lives and experiences. The authors encourage careful planning, including formulating learning objectives, aligning with other curriculum, recruiting appropriate patients, and developing relevant questions to ask the patients. Facilitators, patients and students each need tailored training in the web conference technology and procedures appropriate for their roles in the panel, including for students on appropriate and inappropriate questions and avoiding side conversations in the chat. Two facilitators are recommended, one to guide the conversation with patients and the other to moderate chat, read aloud student questions at appropriate times, and manage any technical issues. Students should dress professionally and turn on cameras at the start and end to wave to patients to welcome and thank them, respectively, but keep cameras and microphones off during the session to minimise bandwidth load and potential distractions. Diverse patients should be recruited, and if required assistance provided to overcome barriers to participation including access to hardware, software, internet and assistive technologies. Faculty implementing or researching patient involvement in health professions education should contemplate how patients can be supported, valued and empowered for greater involvement in the educational community as partners, beyond sharing their stories, and how and why their involvement enhances student learning ( Bennett-Weston *et al*., 2023).

Khurshid and colleagues (2023) provide tips for running the nominal group technique (NGT) using a web conferencing platform (virtual NGT, or vNGT). NGT is a qualitative research technique that uses a ‘structured approach to consensus development and data collection driven by problem-solving, idea inception and prioritisation’ which avoids one or a few participants dominating discussions as can occur in focus groups ( Khurshid *et al*., 2023). Although prompted by COVID-19 lockdowns, the online NGT format proved more efficient than the in-person format, avoiding the need for participants to travel to a single location. Social media was used for recruitment and an online scheduling tool to select the meeting date and time. Web conference software enables audio-visual recording for researchers to review the session later, automatic transcription, screensharing for sign-posting NGT phases and rules, in-built whiteboard or screensharing a word processing program to scribe and later order ideas by theme, breakout rooms or private chat for individual discussion with the facilitator if required, count-down timer to monitor time for NGT phases, and in-built polling or screensharing other polling software for the ranking/voting phase. The authors recommended group sizes of 3–7 for vNGT rather than the usual 6–8 for NGT and note that access to technology and good internet connection may limit participation of persons from disadvantaged backgrounds. Teachers could potentially use vNGT to gather student feedback on their TEL designs for research or evaluation, using a facilitator not involved with the course for anonymity of participants. While vNGT is a research method rather than a pedagogy/andragogy, one could imagine adapting the approach as a learning activity in a problem-based or case-based learning context. For example, students could silently generate possible differential diagnoses or a list of topics for further investigation related to a case. Small groups of students would then share one potential diagnosis or topic at a time in round robin format until no new diagnoses or topics are presented, with a facilitator or one of the students serving as a scribe. Students then work together to arrange similar diagnoses or topics together, and then anonymously vote to rank the list, with further discussion to follow.

Peacock and colleagues (2023) provide several examples of potential uses of ChatGPT 3.5, a large language model or generative artificial intelligence (genAI) that was released 30 November 2022 by OpenAI that presently remains free to use, in medical education across development, teaching, learning and research. The authors point out that careful crafting and refining of prompts is required to obtain relevant outputs, which still require critical evaluation for accuracy and biases and awareness that ChatGPT 3.5 was trained on data to 2021. Transcripts of prompts and ChatGPT outputs are provided as supplementary data. Many of the examples given are for summarising information, providing ideas that can be considered further, and generating drafts that can be refined by the user. Possible uses presented include using ChatGPT to summarise information, articles, or data sets; generating draft proposals, curricula, syllabi, cases for TBL or problem-based learning, checklists for simulation, practice questions and feedback, teaching and learning activities supported by educational theories, and personalised learning plans for students; and suggesting potential solutions to challenges in teaching and practice. The authors also suggest uses in research including reviewing or revising written work, performing thematic analysis on data, developing statements to guide screening for literature reviews, or generating reference lists. While the authors note that ChatGPT 3.5 frequently generates fictitious or irrelevant references, should be used ethically and safely, and output carefully evaluated, edited and further developed by humans, many potential uses described require educators’ careful consideration of university and journal publisher policies. Ethical and legal issues related to genAI in education and academic publishing include bias ( Masters, 2023), potential copyright infringement ( van de Ridder *et al*., 2023) and authorship ( Ellaway & Tolsgaard, 2023). These and other issues require ongoing consideration as the capabilities of genAI grow.

There are many other articles in *MedEdPublish* beyond the TEL collection that address the use of technology to enhance learning in health professions education, prior to the COVID-19 pandemic, during pandemic lockdowns that necessitated emergency remote teaching, and emerging from the pandemic with increased use of online, blended and hybrid learning modes. Technologies that are being increasingly investigated for health professions education include virtual, augmented and mixed reality, artificial intelligence, robotics, serious games and gamification, and open educational resources ( Frenk *et al*., 2022). In-depth evaluation ( Allen *et al*., 2022) and research reporting the implementation by teachers of technologies in various learning designs and environments, and their impacts on health professions students’ learning, enable readers of *MedEdPublish* and other journals to consider the transferability of the findings of educational scholarship to their own teaching contexts.
